# Motion compensated carotid MRI using FID navigators

**DOI:** 10.1186/1532-429X-15-S1-P242

**Published:** 2013-01-30

**Authors:** Petter Dyverfeldt, Vibhas S Deshpande, Tobias Kober, Gunnar Krueger, David Saloner

**Affiliations:** 1 Radiology & Biomedical Imaging, University of California San Francisco, San Francisco, CA, USA; 2 Medical and Health Sciences, Linköping University, Linköping, Sweden; 3 Siemens Medical Solutions USA, Inc., San Francisco, CA, USA; 4 Advanced Clinical Imaging Technology, Siemens Healthcare Sector IM&WS S, Lausanne, Switzerland; 5 Veterans Affairs Medical Center, San Francisco, CA, USA

## Background

Multicontrast-weighted carotid MRI is promising for determination of vulnerability of carotid plaques. However, motion artifacts due to swallowing, breathing, head motion, etc. frequently result in non-diagnostic image quality. The aim of this study was to validate prospective free induction decay (FID) based navigator gating for suppression of motion artifacts in carotid MRI.

## Methods

A prospective FID-navigator comprising a low-flip angle sinc-pulse followed by an ADC readout was implemented in a conventional 2D/3D TSE sequence on a 1.5T scanner (Siemens Avanto) equipped with an 8-element carotid coil. Real-time navigator processing based on that described by Kober et al [2] delivered accept/reject-and-reacquire decisions to the sequence and visual feedback to the scanner user-interface.

7 volunteers were scanned with pulse-gated 2D T2-weighted TSE imaging (pixel size 0.5x0.5 mm2, slice thickness 2 mm, TE = 61 ms, TR = 2 cardiac cycles, bandwidth = 230 Hz/pixel, echo train length = 15). Five scans were performed at a standardized slice location proximal to the carotid bifurcation: One reference scan with volunteers instructed to abstain from swallowing and to breathe shallowly, and two pairs of non-gated and gated scans with volunteers instructed to a) swallow and b) take quick deep breaths at 20, 40, 50, 60, and 80% of the scan.

Two blinded reviewers graded the quality of bilateral carotid artery wall depiction using a 4-point scale. Scores were compared using a paired two-tailed Wilcoxon signed rank test. Moreover, lumen-to-wall sharpness was quantified using a previously described method [3]. Sharpness for the reference, gated, and non-gated scans was compared using a paired two-tailed Student's t-test. Additionally, the effects of swallowing vs. heavy breathing in non-gated images were compared using both image quality metrics. P-value less than .05 were considered significant.

## Results

**Figure 1:**
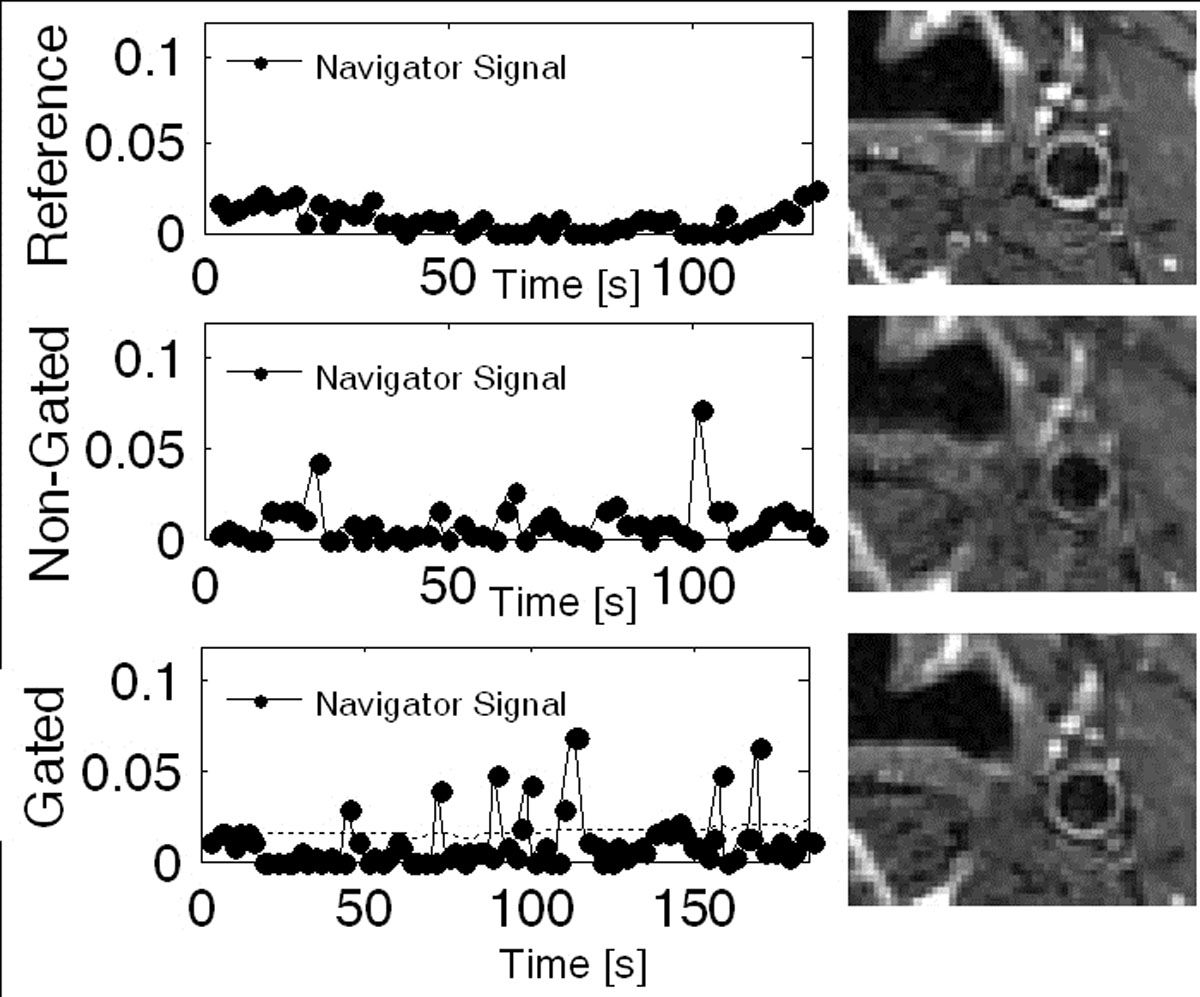
Navigator signal (left) and T2-weighted TSE image (right) for reference (top), non-gated breathing (middle), and gated breathing (bottom) scans. The dashed line in the bottom left panel represents the gating threshold.

**Figure 2:**
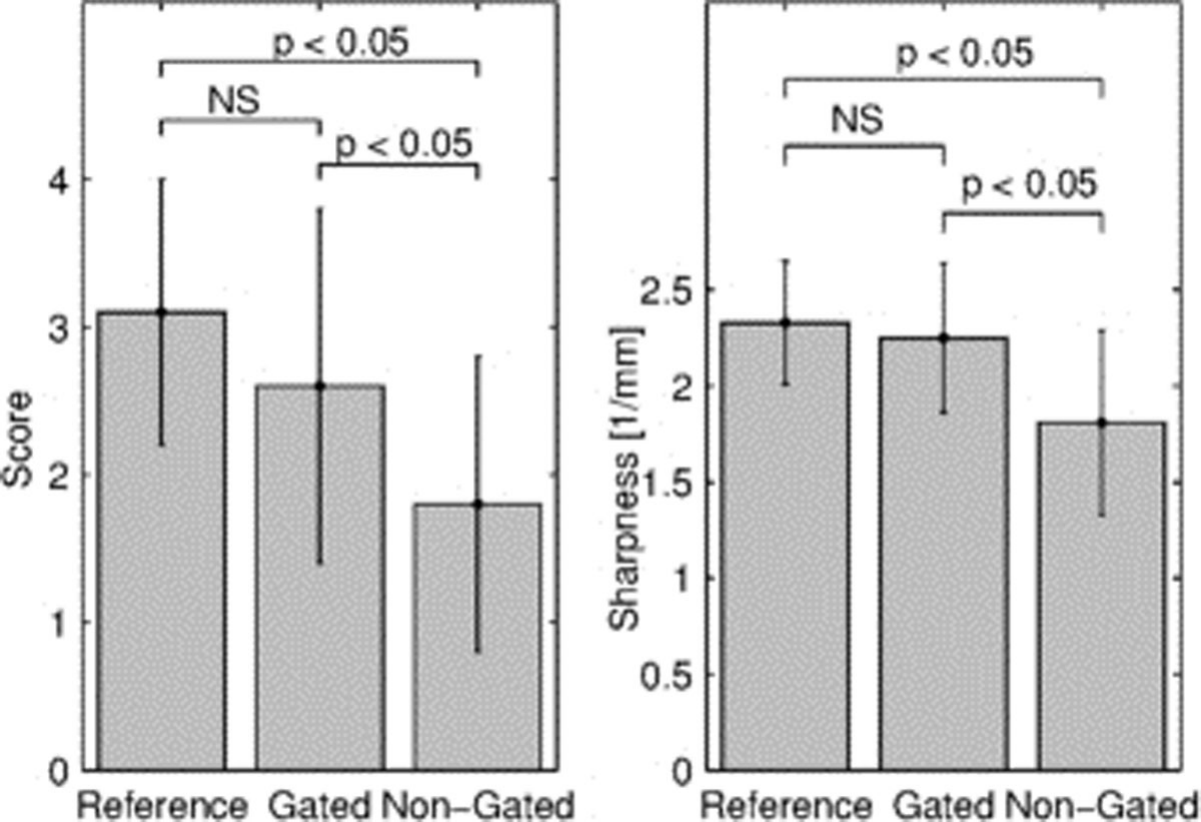
Summary of qualitative and quantitative image quality comparisons. Image quality score (mean ± standard deviation) was 3.1 ± 0.9, 2.6 ± 1.2, and 1.8 ± 1.0 for Reference, Gated, and Non-Gated images respectively. Lumen-to-wall sharpness was 3.1 ± 0.9, 2.6 ± 1.2, and 1.8 ± 1.0 for Reference, Gated, and Non-Gated images respectively.

FID navigator signals and images from one subject are shown in Figure 1. Results from the image quality analysis are summarized in Figure 2. Artifacts caused by the employed motion tasks deteriorated image quality in the non-gated scans. These artifacts were alleviated with the proposed FID-navigator. There was no difference in vessel wall sharpness or image quality score between the reference and gated images. For images acquired during motion, both the vessel wall sharpness and the image quality score were higher in the gated compared to non-gated images. There was no difference between swallowing and heavy breathing.

## Conclusions

FID-navigator gating successfully alleviates motion artifacts in carotid MRI. The additional finding that there was no difference in image quality between data acquired during heavy breathing and swallowing indicate that breathing is an underestimated cause of artifacts in carotid MRI.

## Funding

Fulbright Commission

Swedish Heart-Lung Foundation

Swedish Brain Foundation
